# ﻿*Neotriniakurramica* (Poaceae, Stipeae), a new species from Parachinar (Khyber Pakhtunkhwa, Pakistan)

**DOI:** 10.3897/phytokeys.253.145562

**Published:** 2025-03-14

**Authors:** Amir Sultan, Amjad Khan, Raees Khan, Asif Mehmood, Murtaza Hussain, Marcin Nobis

**Affiliations:** 1 National Herbarium of Pakistan (Stewart Collection), National Agricultural Research Centre, Pakistan Agricultural Research Council, Islamabad, Pakistan National Agricultural Research Centre, Pakistan Agricultural Research Council Islamabad Pakistan; 2 Kunming Institute of Botany, Chinese Academy of Sciences, Kunming, Yunnan, China Kunming Institute of Botany, Chinese Academy of Sciences Kunming China; 3 Department of Botany, Kohat University of Science and Technology, Kohat, Pakistan Kohat University of Science and Technology Kohat Pakistan; 4 Department of Botany, Government Postgraduate College, Parachinar, Pakistan Department of Botany, Government Postgraduate College Parachinar Pakistan; 5 Institute of Botany, Faculty of Biology, Jagiellonian University, Kraków, Poland Jagiellonian University Kraków Poland

**Keywords:** Lemma micromorphology, Pakistan, Stipeae, taxonomy

## Abstract

The family of grasses (Poaceae) is one of the most diverse plant families, with the tribe Stipeae representing an ecologically significant group of more than 600 species primarily distributed in arid and semi-arid regions. The genus *Neotrinia*, a lesser-known member of this tribe, is characterised by unique morphological traits, including distinctive patterns of lemma epidermis. During field studies in the upper Kurram Valley, Pakistan, we found a new species, *Neotriniakurramica*, which is described here. This perennial grass exhibits distinct morphological features, that differentiate it from previously known species. It is characterised by having up to 8.5 mm long ligules of cauline leaves, up to 15 cm long panicles bearing up to 41 spikelets, subequal glumes, lemma bearing two apical lobes, palea almost equal to lemma, 0.9–1.2 mm long obtuse callus and 50–70 mm long scabrid awns. Figures illustrating the new taxon and a comparison of distinguishing characters of the species representing the genus *Neotrinia* are presented.

## ﻿Introduction

The family Poaceae is among the most diverse and ecologically important plant families, encompassing more than 789 recognised genera (Soreng et al. 2022). They dominate diverse ecosystems ranging from tropical savannas and temperate grasslands to croplands (Hodkinson and Parnell 2007). Within Poaceae, the tribe Stipeae represents ecologically significant lineage comprising more than 600 species distributed across 34 genera (Soreng et al. 2022). Members of Stipeae are primarily distributed in arid and semi-arid regions, where they play critical roles in stabilising soils, providing forage, and maintaining ecological balance. The tribe is characterised by its unique morphology, including distinctive awned florets and complex seed dispersal mechanisms. The evolutionary history of Stipeae suggests an origin in Eurasia with Central Asia as its species diversity hotspot, with subsequent diversification influenced by climate shifts and habitat specialisation (Nobis et al. 2019, 2020; Vintsek et al. 2022; Krzempek et al. 2024; Sinaga et al. 2024).

The genus *Neotrinia*, is a relatively little-known member of tribe Stipeae, established to accommodate species with specific morphological traits that differentiate them from closely related genera such as *Stipa*, *Achnatherum*, *Piptatherum*, *Ptilagrostis*, *Stipellula* or *Trikeraia* (Nobis et al. 2019, 2020, Peterson et al. 2019). The most characteristic features of the members of *Neotrinia* are as follows: plants rather tall, from 50 to 250 cm high, densely tufted, panicle axis and branches covered by very dense short hairs, glumes abaxially covered by sparsely distributed and short prickles, lemma with two permanent apical unawned lobes (tithes) terminated by persistent, uni-geniculate or indistinctly bent scabrous awn, from 6 to 70 mm long, lemma epidermal pattern with elongated fundamental (long) cells (several times longer than wide) with deeply sinuous side walls; silica bodies rounded with adjacent cork cells and sparse and scattered hooks, prickles and macrohairs scattered throughout the entire lemma surface (Nobis et al. 2019; Nobis et al. 2020).

To date, only two species were included in the genus *Neotrinia*, *N.splendens* (Trin.) M. Nobis, P.D. Gudkova & A. Nowak and *N.chitralensis* (Bor) M. Nobis. *Neotriniasplendens* has a rather wide distribution range (from southern Russia throughout Mongolia, China, Japan, Kazakhstan, Kyrgyzstan, Tajikistan, Uzbekistan, Turkmenistan, Afghanistan, Iran, and Pakistan up to India (Nobis et al. 2019) and holds particular interest in its adaptations to harsh environments. Whereas *N.chitralensis* is treated as an endemic to Chitral Mts in Pakistan (Cope 1982). During field studies conducted in Khyber Pakhtunkhwa (upper Kurram Valley, western Pakistan), we found interesting specimens representing the tribe Stipeae, and described below as a new species belonging to the genus *Neotrinia*. This new taxon exhibits unique morphological characters that distinguish it from previously described species within the genus. Its discovery highlights the rich but under-documented biodiversity of the region and emphasises the need for continued botanical exploration in the mountains of Asia.

## ﻿Methods

During recent field expeditions carried out in Parachinar (upper Kurram valley) in April-May, 2024 by Amir Sultan, Amjad Khan and Murtaza Hussain, interesting specimens of *Neotrinia* were collected from Malakyar Tangay area of Zerhan. Herbarium specimens of this species have been deposited at RAW and KRA (herbarium codes follow Thiers 2025). Morphological revision of herbarium material of all species representing the genus *Neotrinia* and allies, preserved at BM, CAL, COLO, E, K, KRA, KUN, LE, M, MO, MSB, PE, W herbaria was carried out by Marcin Nobis.

Micromorphological details of the lemma epidermis, leaf surfaces and awn were studied directly under both a stereomicroscope (Olympus 605371; Tokyo, Japan), JEOL-5910 scanning electron microscope (Tokyo, Japan) installed at the Centralized Resource Laboratory Department of Physics, University of Peshawar and Hitachi S-4700 scanning electron microscope at Jagiellonian University in Krakow. The dried material was coated with gold and then photographed under various magnifications.

## ﻿Taxonomy

### ﻿ Neotrinia kurramica

Taxon classificationPlantaePoalesPoaceae

A.Sultan, M.Nobis & Amjad Khan, sp. nov.

2CEDCD5D-2D22-5876-AF97-176821D711E1

urn:lsid:ipni.org:names:77358342-1

Figs 1–3

#### Type.

Pakistan • Khyber Pakhtunkhwa, Upper Kurram Valley, Parachinar, Zerhan, Malakyar Tangai above Mulla Bagh, grassland on calcareous rocks, 33°56'47.4"N, 70°10'03.4"E, 2220 m, 6 May 2024, *Amir Sultan, Amjad Khan & Murtaza Hussain* s.n. (***holotype*** RAW [barcode 103365], ***isotype*** KRA00639009).

#### Description.

**Figure 1.**
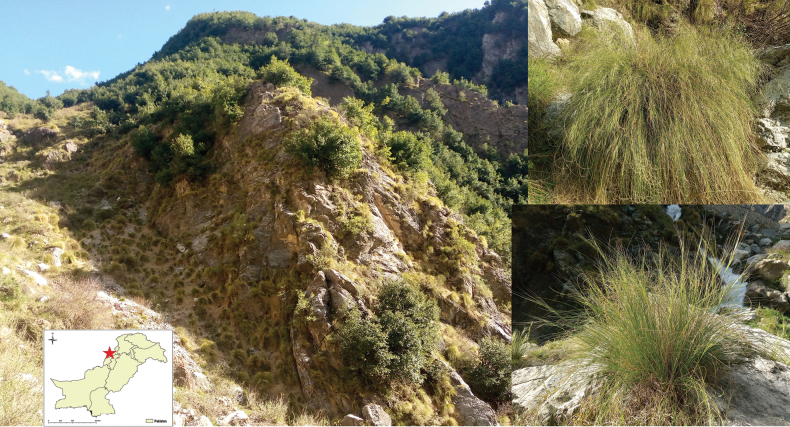
Habitat and general habit of *Neotriniakurramica* in northwestern Pakistan.

**Figure 2.**
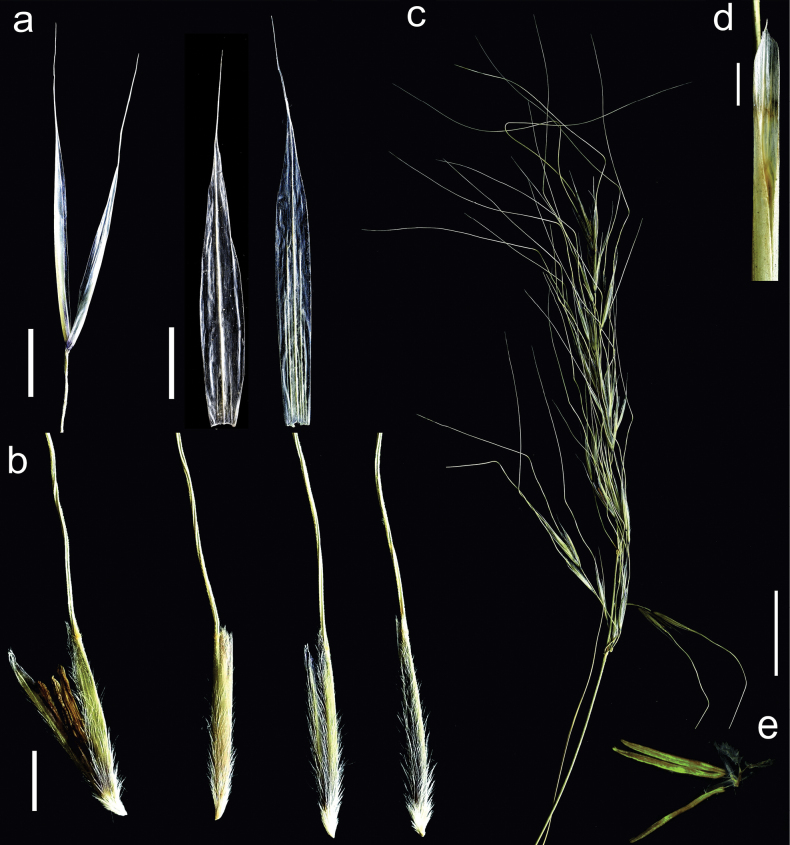
Main morphological characters of *Neotriniakurramica*: glumes (**a**), florets (**b**), panicle (**c**), ligule of culm leaf (**d**), generative elements of flower, three anthers and two styles of ovary (**e**). Scale bars: 3 mm (**a, b, d**); 2 cm (**c**).

**Figure 3.**
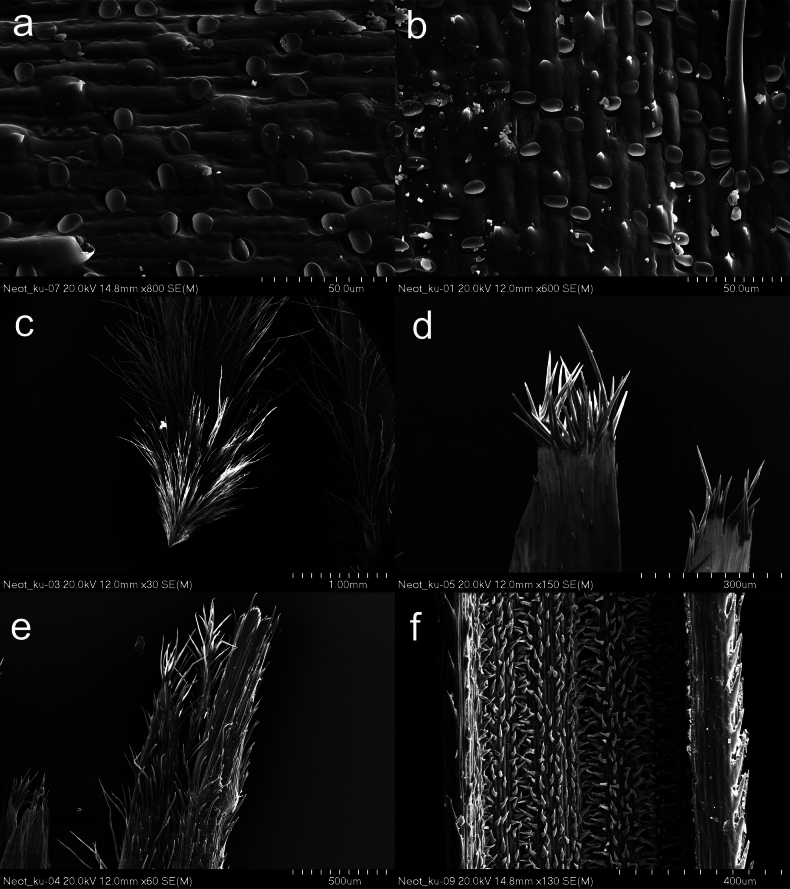
Micromorphological characters of *Neotriniakurramica*: abaxial surface of lemma (**a, b**), callus (**c**), palea apex (**d**), lemma lobes (**e**), indumentum of the adaxial surface of leaf (**f**).

Plant perennial, densely tufted with numerous culms and vegetative shoots (Fig. 1). ***Culms*** 40–80 cm tall, with 2 nodes, nodes pilose or pubescent. ***Leaves of vegetative shoots***: sheaths glabrous to sparsely and shortly pilose with white edge, scabrid at margins; ligules membranous, 2–3.5 mm long, acute to acuminate, apex shortly ciliate, and pubescent on the back; blades convolute, pale green, 20–55 cm long, 0.6–0.8(–1.0) mm in diameter, adaxial surface covered by dense and up to 0.15 mm long hairs (Fig. 3f), while abaxial surface glabrous to minutely scabrous along the midrib. ***Cauline leaves***: lower sheaths scabrous whereas the uppermost glabrous to minutely scabrous, the margins white and sparsely pubescent; ligules 3.5–8.5 mm long, acute or acuminate, at apex and the back shortly ciliate (Fig. 2d); blades convolute, pale green, up to 25 cm long, adaxial surface densely covered by short hairs, while abaxial surface glabrous to minutely scabrid along the midrib and margins. ***Panicle*** 10–15 cm long, contracted, with (15–)29–41 spikelets, exserted (sometimes lower branches enclosed by sheath of upper cauline leaf), branches erect (basal branches often spreading), hirsute with up to 0.5 mm long hairs, lower ones in threes (apical branches single, paired or sometimes up to 4 branches per node), the lower ones up to 18 mm long (Fig. 2c). ***Glumes*** subequal, pale green with hyaline membranous margins and at the top taper into a long hyaline tip, lower glume 14–15 mm long, three nerved, somewhat shorter than the upper glume, which is 5–7 nerved, lanceolate, 15–16 mm long, abaxially sparsely covered by scattered short prickles (Fig. 2a). ***Floret*** (anthecium = callus + lemma) 9–10.5 mm long (including apical lobes of lemma), ca. 1 mm wide (Fig. 2b). ***Callus*** 0.9–1.2 mm long, densely and long-pilose, hairs 0.5–1 mm long; peripheral ring 0.1–0.25 mm in diameter elliptic, scar circular (Fig. 3c). ***Lemma*** pale green to purple, dorsal surface with elongated basal (long) cells, rounded silica bodies with adjacent cork cells and sparse and scattered hooks, prickles and ascending hairs 0.75–1 mm long (Fig. 3a, b); apical lobes of lemma 0.6–1.0 mm long and covered by short hairs, the top of the lemma with 0.15–0.2 mm long hairs forming corolla (Figs 2b, 3e). ***Awn*** 50–70 mm long, unigeniculate; column 18–21 mm long, twisted, straw-coloured, covered by 0.2 mm long hairs, gradually decreasing in length towards geniculation; seta straight 30–50 mm long, hairs in lower part of seta 0.1–0.2 mm long, gradually decreasing in length towards apex. ***Palea*** equal to lemma in length, with a dorsal line of hairs and a ring of short hairs at the apex (Fig. 3d). ***Anthers*** yellow, glabrous, 6 mm long, filaments ca. 1 mm long (Fig. 2e). ***Lodicules*** 3, linear-lanceolate, 2–2.4 mm long, 0.3 mm wide. ***Ovary*** with 2 styles (Fig. 2e). ***Caryopsis*** not seen.

#### Distribution and ecology.

*Neotriniakurramica* is so far only known from the type locality in Upper Kurram, Khyber Pakhtunkhwa Province (Fig. 1), occurring at an elevation of about 2220 m. The population of *N.kurramica* was found growing along stony/gravelly slopes leading to the edges of a mountain stream. While these slopes are dominated by grasses, oak (*Quercusbaloot* Griff.) forests dominate the hilltops above these slopes. *Neotriniakurramica* grows in association with *Isodonrugosus* (Wall. ex Benth.) Codd, *Duthieaoligostachya* (Munro ex Aitch.) Stapf, *Sophoramollis* (Royle) Graham ex Baker, *Piptatherum* sp. and *Cirsiumfalconeri* (Hook. f.) Petr. It is expected to have a distribution in similar habitats of Upper Kurram valley. Further explorations in the area are needed, to determine its population size, distribution range and to evaluate conservation status of this new species.

#### Paratype.

Pakistan • Khyber Pakhtunkhwa, Upper Kurram, Parachinar, Zerhan, Malakyar Tangay above Mulla Bagh, 22 May 2021, *Amir Sultan, Asif Mehmood, Waqar Hassanain & Noor Ali Shah* s.n. (RAW [barcode 101893]).

#### Etymology.

The name of the new species originates from Kurram Valley.

#### Similar species.

*Neotriniakurramica* differs significantly from all the remaining species representing the genus by having awns 50–70 mm long vs. 5–12 or 16–21 mm long in *N.splendens* and *N.chitralensis* respectively (Table 1). The new species is slightly similar also to *Achnatherumjacquemontii*, however differs in having longer lemmas, longer glumes, longer awns, and completely different patterns of the lemma morphology sow-like vs. maize-like respectively (cf. Nobis et al. 2020).

**Table 1.** A comparison of the main characters distinguishing *Neotriniakurramica* from other members representing the genus occurring in Pakistan.

Character	*N.kurramica*	*N.chitralensis*	*N.splendens*
Floret (anthecium)	9–10.5 mm	9–11 mm	4.0–7.2 mm
Apical lemma teeth	0.6–1.0 mm, pilose	2–2.5 mm, glabrous	0.5–1.2 mm, pilose
Callus	0.9–1.2 mm	0.5–0.6 mm	0.3–0.5 mm
Awn	50–70 mm	16–21 mm	5–12 mm
Glumes	14–16 mm long, subequal, the lower slightly shorter than the upper, at the top tapering into long hyaline tips	9.5–12 mm long, subequal, the lower slightly shorter than the upper, at the top without long hyaline tips	5–8.5 mm long, lanceolate, distinctly unequal, the lower 1–1.7 mm shorter than the upper, at the top without long hyaline tips

### ﻿A key to genera of the tribe Stipeae in Pakistan

**Table:** 

1	Lemma lobes awn-like, 2–3 mm long, setaceous	***Trikeraia***
–	Lemma without awn-like lobes, lobes (if present) usually less than 2.5 mm long	**2**
2	Surface of lemma epidermis covered by numerous and rounded silica bodies, basal cells as wide as long or wider than longer, hooks usually absent, at least in the middle part of lemma (lemma epidermal pattern maize-like)	**3**
–	Surface of lemma epidermis covered with square, rectangular or elongated basal cells (2–11 times longer than wider), silica bodies usually associated with cork cells, hooks usually present and more or less densely distributed (lemma epidermal pattern sow-like)	**4**
3	Plants annual	***Stipellula***
–	Plants perennial	***Achnatherum***
4	Awns straight, scabrous. Floret (anthecium) usually dorsally compressed. Callus short, up to 0.3 mm long	***Piptatherum***
–	Awns geniculate, scabrous or variously pilose. Floret (anthecium) terete or laterally compressed. Callus longer than 0.3 mm	**5**
5	Callus stiff, usually acute at the apex, (0.5–)1–4(–6) mm long. Lemma epidermis with square or rectangular cells associated with numerous hooks (visible under high magnification) and reniform to ovate silica bodies	***Stipa***
–	Callus blunt, usually rounded at the apex, (0.3–)0.5–1(–1.2) mm long. Lemma epidermis with elongated cells 4–11 times longer than wider, associated with ovate or elongated silica bodies and rather sparse or absent, at least in the middle part of the lemma, hooks (visible under high magnification)	**6**
6	Glumes glabrous or with sparse cilia along the middle vein, lower segment of awn pilose, with hairs over 0.3 mm long. Surface of lemma epidermis with elongated, 1–3-constricted silica bodies, without or with very sparse hooks	***Ptilagrostis***
–	Glumes scabrous or densely covered by short prickles, lower segment of awn scabrous, with prickles up to 0.3 mm long. Surface of lemma epidermis with ovate to rounded silica bodies and sparse hooks	***Neotrinia***

## Supplementary Material

XML Treatment for Neotrinia kurramica

## ﻿Additional information

### Author ORCIDs

Amir Sultan https://orcid.org/0000-0003-2116-9502

Amjad Khan https://orcid.org/0000-0002-5337-7237

Raees Khan https://orcid.org/0000-0003-1388-7141

Asif Mehmood https://orcid.org/0009-0009-3227-2522

Murtaza Hussain https://orcid.org/0009-0005-7339-7832

Marcin Nobis https://orcid.org/0000-0002-1594-2418

### Data availability

All of the data that support the findings of this study are available in the main text.
